# Prevention of Vascular Dysfunction after Preeclampsia: A Potential Long-Term Outcome Measure and an Emerging Goal for Treatment

**DOI:** 10.1155/2012/704146

**Published:** 2011-12-08

**Authors:** Merzaka Lazdam, Esther F. Davis, Adam J. Lewandowski, Stephanie A. Worton, Yvonne Kenworthy, Brenda Kelly, Paul Leeson

**Affiliations:** ^1^Department of Cardiovascular Medicine, Oxford Cardiovascular Clinical Research Facility, University of Oxford, John Radcliffe Hospital, Oxford OX3 9DU, UK; ^2^Department of Obstetrics and Gynaecology, University of Oxford, John Radcliffe Hospital, Oxford OX3 9DU, UK

## Abstract

Preeclampsia is increasingly being recognised as more than an isolated disease of pregnancy. In particular, preeclampsia has emerged as an independent risk factor for maternal cardiovascular disease and has recently been recognised as a risk factor for cardiovascular disease in children exposed in utero. Preeclampsia and cardiovascular disease may share important pathophysiological and molecular mechanisms and further investigation into these is likely to offer insight into the origins of both conditions. This paper considers the links between cardiovascular disease and preeclampsia and the implication of these findings for refinement of the management of patients whose care is complicated by preeclampsia.

## 1. Introduction

Although traditionally preeclampsia has been viewed as a condition that resolves completely with the delivery of the placenta, there is now increasing evidence that preeclampsia may constitute a condition with significant long-term health implications for both the mother and child. In particular preeclampsia has recently emerged as an independent risk factor for maternal cardiovascular disease 10–15 years after the index pregnancy [1–3]. A history of preeclampsia is therefore now considered a relevant factor in the cardiovascular risk assessment in women [4] and is associated with an increase in risk similar in magnitude to a history of dyslipidemia [5]. Meta-analysis has demonstrated that in the 10–15 years following a preeclamptic pregnancy women have an increased risk of developing hypertension (RR 3.7 95% C.I. (2.7–5.05, *P* < 0.001)), coronary artery disease (RR 2.16 95% C.I. (1.86–2.52, *P* = 0.001)), and stroke (RR 1.81 95% C.I. (1.45–2.27, *P* = 0.00) [1]. Additionally, it has been demonstrated that there is a graded relationship between the risk of cardiac disease and the severity of preeclampsia with maternal risk being greatest with early onset or severe preeclampsia [6]. Children born to preeclamptic women have also been demonstrated to have elevated blood pressure in childhood and adolescence [7–12]. 

This highlights potentially important pathophysiological or molecular links between preeclampsia and cardiovascular disease. Greater insight into these links may identify new opportunities to understand disease predisposition and treatment and may also raise the possibility that prevention of vascular dysfunction should be an important long-term goal of preeclampsia management. This paper will consider the current literature considering the links between cardiovascular disease and preeclampsia and the implication of these findings for refining the management of patients whose care is complicated by preeclampsia.

## 2. What Links Preeclampsia and Cardiovascular Disease?

The aetiology of preeclampsia remains incompletely understood, though disturbed placentation and placental functioning in early pregnancy remains the leading hypothesis [31]. During normal placental development, fetal cytotrophoblasts invade the maternal spiral arteries transforming them to high-caliber capacitance vessels providing low resistance placental perfusion adequate to sustain fetal growth [32]. However, inadequate spiral artery remodeling in preeclampsia is thought to lead to chronic placental ischaemia or intermittent flow through the narrow muscular arteries thereby creating an ischaemia-reperfusion phenomenon [33].

Reactive oxygen species and cytokines released from the ischaemic placenta trigger a systemic oxidative stress [34] and contribute to the exaggerated systemic inflammatory reaction in preeclampsia [33, 35]. Syncytiotrophoblasts undergoing apoptosis also shed increased numbers of microparticles in the maternal circulation which contribute to this process. The release of cytokines and acute phase proteins, such as TNF*α*, leptin, and PAI-1, not only enhance the inflammatory response but also induce some of the observed metabolic disturbances in preeclampsia including insulin resistance, lipolysis, and hyperlipidaemia [33, 34].

**Figure 1:**
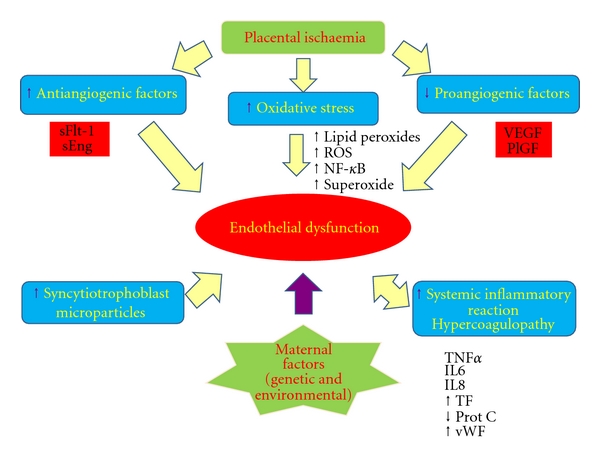
Molecular and vascular mechanisms of endothelial dysfunction in preeclampsia. Defective placentation, a common feature of preeclampsia, triggers a cascade of events including oxidative stress and exaggerated inflammatory reaction and angiogenic imbalance which exacerbate endothelial dysfunction. Impaired endothelial function plays a central role in the clinical manifestations of preeclampsia such as hypertension and proteinuria.

Furthermore, hypoxic oxidative stress and inflammatory stimuli provoke the release of antiangiogenic factors (via NF-*κ*B pathways) [33, 36–38]. Soluble Fms-Like Tyrosine kinase-1 (sFLT-1), a circulating truncated form of VEGF receptor, binds and reduces levels of VEGF and PlGF in the maternal circulation thereby inhibiting angiogenesis and vasodilatation [39]. This aberrant vasculature is thought to lead to a cascade of events which end in symptomatic systemic endothelial dysfunction [40]. Soluble endoglin, a TGF-*β* coreceptor, is one of the antiangiogenic factors that enhances vascular permeability and may possibly affect nitric oxide synthesis and vasodilatation via altered downstream signalling in the TGF-*β* pathway contributing to the endothelial dysfunction [35] (Figure 1).

**Figure 2:**
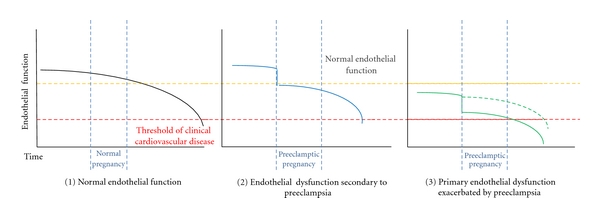
Theoretical timelines of impairment of endothelial function and development of cardiovascular disease following preeclamptic pregnancy. (1) In the normal individuals there is a gradual age-related reduction in endothelial function, which can be exacerbated by the presence of cardiovascular risk factors and associates with the future risk of clinical cardiovascular disease. (2) Women who experience preeclamptic pregnancies are known to have impaired endothelial function during pregnancy and up to 3 years following an affected pregnancy. It is possible that these women begin life with normal endothelial function, which is acutely impaired during a preeclamptic pregnancy. This followed by ongoing age-related decreases in endothelial function may relate to the increased incidence of cardiovascular disease in these individuals. (3) Alternatively, women who develop preeclampsia may have primary endothelial dysfunction which both puts them at risk of preeclampsia, this may then be exacerbated by the preeclamptic pregnancy (solid line), or simply persist (dotted line), in either case leading to higher incidence of cardiovascular disease.

Consistent with these biological changes, reduced endothelial-dependent vasodilatation in conduit and resistance arteries [21, 41–47] has been demonstrated in women who have preeclampsia. They also appear to have increased arterial stiffness [13], increased atherosclerosis [48], and diminished capillary density [49]. Biochemical markers of endothelial activation and dysfunction are also elevated in preeclampsia [34, 50]. Maternal endothelial dysfunction is also present in conduit vessels before the onset of clinical disease [42] and up to three years after an affected pregnancy [24, 51, 52]. The increase in cardiovascular risk following preeclampsia may be a consequence of the pro-atherogenic impact of persistent endothelial dysfunction, a result of subclinical endothelial injury at the time of pregnancy, or to preexisting differences in endothelial function that predispose to both conditions (Figure 2).

Dysfunction of the vascular endothelium is a key factor in the development of atherosclerotic cardiovascular disease and has been demonstrated to precede clinically identifiable structural changes in the vasculature [53]. Peripheral dysfunction of the vascular endothelium has been demonstrated to correlate with increased risk of clinical events [54] and all cause mortality [55] and associates with many traditional cardiovascular risk factors including hypertension, diabetes mellitus, insulin resistance, hypercholesterolemia, and smoking [56]. In addition to this, endothelial dysfunction has been demonstrated in young adults predisposed to hypertension without clinical evidence of arterial disease [57]. Endothelial health and nitric oxide synthase activity are crucial in modulating arterial distensibility [58–60] and carotid intima media thickness [61, 62] independent of risk factors [62] as well as myocardial hypertrophic responses in animals [63–65]. Left ventricular mass, another powerful independent predictor of mortality and morbidity in adults free of clinical disease [66], has a graded relationship with vascular endothelial vasomotor responses in hypertensive adults [67–72]. The strong relationship between endothelial dysfunction and cardiovascular outcomes and risk factors make it an interesting pathophysiological endpoint in the evaluation of therapies designed to modify long-term cardiovascular risk.

## 3. Maternal Vascular Function as an Intermediate Endpoint in the Management of Preeclampsia

As women who have experienced preeclampsia constitute a relatively young group, and there is potentially a prolonged period between exposure and clinical outcome, intermediate measures which may indicate a potentially modifiable change in risk are of particular value. Noninvasive measures of vascular function are widely used as surrogate markers of cardiovascular risk [73, 74]. For example, increasing arterial stiffness, demonstrated by an increase in pulse wave velocity, is an antecedent factor in elevated blood pressure, predicts the future cardiovascular risk of adults, and correlates strongly with the presence of atherosclerosis [75]. Similarly, early evidence of atherosclerosis demonstrated by increased thickness of the arterial wall is correlated with coronary artery disease and is predictive of future infarction and stroke [75, 76]. Changes in these parameters may therefore also offer unique insight into the cardiovascular risk of women and children following a preeclamptic pregnancy.

 Several relatively small-scale studies have also demonstrated vascular dysfunction after pregnancy complicated by preeclampsia with evidence of endothelial dysfunction in the macrocirculation [14, 16, 18, 22–24, 26] up to a median of 3 years and the microcirculation up to 25 years later [15, 27, 30], as well as increased arterial stiffness [13, 14, 16, 17] up to almost 5 years after the index pregnancy and atherosclerosis over 3 months postpartum [19]. Elevation of systemic biomarkers of endothelial injury and inflammation have also been described between 6 weeks [77] and 20 years [78–80] following preeclampsia. The characteristics and results of current literature considering the impact of preeclampsia on vascular structure and function are summarised in Table 1. There is, however, some disparity in results with some studies demonstrating no change in vascular function following preeclampsia [21, 25, 28, 81]. This may in part reflect heterogeneity in patient cohorts, severity of preeclampsia, size of cohorts, the timing of the studies following the affected pregnancy, as well as differences in the vascular beds studied and the techniques employed. In the future more detailed long-term follow-up studies of such women may clarify changes in cardiovascular physiology and inform future treatment.

## 4. Offspring Vascular Function Following Preeclampsia

Even more than their mothers, children born following a preeclamptic pregnancy, constitute a cohort where early life preventative strategies may have a profound impact on future cardiovascular risk. Furthermore, adults whose mothers had preeclampsia themselves have a higher risk of the condition. Therefore, in this group evidence of subtle changes in vascular physiology indicating changes in risk are of particular importance. The body of literature considering cardiovascular outcomes in the offspring of preeclamptic pregnancies is sparse when compared to that considering maternal health. A single 60-year-follow-up study of individuals born to preeclamptic women demonstrated an increased risk of stroke in later life (RR 1.9 95% C.I. (1.2–3.0, *P* = 0.01)) [82]. Offspring of hypertensive pregnancies have also been shown to have an increased risk of hypertension as adults [82, 83] as well as having increased, although not pathological, blood pressure in childhood and adolescence [84]. Meta-analysis suggests that the magnitude of this increase in young individuals for systolic blood pressure is 2.3 mmHg and for diastolic blood pressure is 1.7 mmHg [84]. Although such increases are unlikely to be clinically recognisable, it may have significant public health significance as a 2 mmHg rise in systolic blood pressure has been associated with a 7% increase in ischaemic heart disease mortality and a 10% increase in stroke [85].

Preterm offspring born to hypertensive pregnancies demonstrate a distinct cardiovascular phenotype, characterised by reduced conduit artery endothelial function and increased evidence of early atherosclerosis, compared to individuals born preterm to normotensive women [7]. A finding which has now been replicated in other groups [27, 86, 87] and may be similar to the vascular dysfunction seen in women following preeclamptic pregnancies [27]. Although the underlying mechanisms remain unknown, there is substantial evidence of “programming” of aspects of vascular biology during fetal development, in particular endothelial responses [88] and arterial stiffness [89]. The risk to the offspring is likely to be mediated through changes in maternal blood pressure, vascular resistance in the placenta, or exposure to maternal factors (such as antiangiogenic factors [90], vasoactive substances [91], and reactive oxygen species) in the fetomaternal circulation. Optimal management of preeclampsia may have indirect benefits to reduce cardiovascular risk in the offspring of such pregnancies [92]. Hence, better understanding of the long-term vascular changes in offspring of preeclampsia may allow assessment of novel and previously unrecognised long-term outcomes of preeclampsia with important public health significance.

## 5. Conclusions and Future Directions

It is now becoming clear that preeclampsia is more than an isolated disease of pregnancy. The long-term health implications of this condition for both the women and their children are increasingly being recognised and incorporated into clinical risk assessments [4]. Both women and children exposed to preeclampsia exhibit an adverse vascular phenotype, a propensity to subclinical atherosclerosis, and increased risk of adverse cardiac and vascular events in future life. As preeclampsia affects 2–5% of the population this altered risk is relevant to the health of 1.2 to 3 million people in the UK and 6 to 15 million people in the USA. Optimal management of preeclampsia may be able to improve short and long-term vascular outcomes in these individuals. While we remain unable to effectively prevent preeclampsia attempts to reduce its long-term impact on those exposed are of potential importance. Future studies that define the detailed cardiovascular phenotype of those exposed to preeclampsia may allow identification of potential targets for future preventative strategies. Furthermore, studies into the mechanisms underlying the altered cardiovascular phenotype may provide unique insight into pathophysiological or molecular links between preeclampsia and cardiovascular disease, which may direct us to novel treatment strategies for both conditions. Vascular dysfunction is an early marker of cardiovascular risk, correlating with future risk of cardiac events and preceding structural vascular change [53]. Improvement in vascular function would therefore be a valuable intermediate endpoint in studies aiming to reduce risk in this potentially young and generally asymptomatic population before the onset of clinical disease.

## Figures and Tables

**Table 1:** Studies assessing the long-term impact of preeclampsia on vascular function. AIx (augmentation index) and PWV (pulse wave velocity) are robust surrogate markers of aortic stiffness. Endothelial-mediated vasodilatation was measured in conduit arteries by FMD (flow-mediated dilatation) and in the resistance arteries using VOP (venous occlusion plethysmography) to measure FBF (forearm blood flow) or peripheral arterial tonometry (PAT) to measure RHI (reactive hyperaemic index). Mircovascular endothelial responses were quantified in the microcirculation using LDF (laser Doppler flowmetry) and iontophoresis. SGA refers to small for gestational age.

Study	Subjects (Preeclampsia/control)	Interval after delivery	Vascular measures	Results in women with previous preeclampsia
*Aortic stiffness*				

Robb et al. 2009 [13]	15/22	7 weeks	AIx, PWV	Elevated PWV and AIx.
Yinon et al. 2010 [14]	24/16	6–24 months	AIx	Increased AIx in women with early onset preeclampsia.
Evans et al. 2011 [15]	18/50	6–36 months	PWV	No significant differences in central arterial stiffness.
Páez et al. 2009 [16]	20/20	2 years	PWV, AIx	Elevated PWV and AIx.
Elvan-Taşpinar et al. 2005 [17]	44/46	4–56 months	PWV	Elevated PWV.
Lampinen et al. 2006 [18]	30/21	5-6 years	AIx	No significant differences in AIx.

*Subclinical atherosclerosis*				

Blaauw et al. 2006 [19]	22/22	≥3 months	Femoral and carotid IMT	Increased IMT with early onset preeclampsia.

*Conduit artery endothelial function*				

Kuscu et al. 2003 [20]	15/11	2 and 6 weeks	FMD	Reduced FMD both during pregnancy and postpartum (no control data postpartum).
Noori et al. 2010 [21]	45/21	12 weeks	FMD	No significant differences in FMD compared to controls.
Yinon et al. 2010 [14]	24/16	6–24 months	FMD	Reduced FMD in women with early onset preeclampsia.
Hamad et al. 2007 [22]	18/17	15 ± 3 months	FMD	Reduced FMD and endothelial independent dilatation in women with severe preeclampsia.
Germain et al. 2007 [23]	25/22	16 ± 3.5 months	FMD	Reduced FMD.
Páez et al. 2009 [16]	20/20	2 years	FMD	Reduced FMD.
Chambers et al. 2001 [24]	113/48	3 years median	FMD	Reduced FMD particularly in women with recurrent preeclampsia and recovery of endothelial function with ascorbic acid.

*Resistance artery endothelial function*				

Lommerse et al. 2007 [25]	32/10	0.64–1.6 years	VOP	No significant difference in FBF.
Agatisa et al. 2004 [26]	16/14	9.9 ± 0.5 months	VOP	Reduced endothelium-mediated FBF.
Evans et al. 2011 [15]	18/50	6–36 months	VOP	Reduced FBF in response to mental stress.
Lampinen et al. 2006 [18]	30/21	5-6 yrs	VOP	Significantly reduced FBF to acetylcholine (ACh) and sodium nitroprusside (SNP).
Kvehaugen et al. 2011 [27]	26/17	5–8 years	PAT	RHI comparable between preeclamptic women and controls. Women with SGA baby had significantly lower RHI.

*Cutaneous microvascular function*				

Khan et al. 2005 [28]	15/54	6 weeks	LDF and iontophoresis	No significant differences between women in endothelial dependent or independent microvascular dilatation.
Blaauw et al. 2005 [29]	25/23	7.0 ± 2.8 months	LDF and iontophoresis	Greater microvascular vasodilator responses in preeclampsia.
Ramsay et al. 2003 [30]	10/10	16–25 years	LDF and iontophoresis	Reduced response to endothelial-dependent and -independent dilatation.

## References

[1] Bellamy L, Casas JP, Hingorani AD, Williams DJ (2007). Pre-eclampsia and risk of cardiovascular disease and cancer in later life: systematic review and meta-analysis. *British Medical Journal*.

[2] McDonald SD, Malinowski A, Zhou Q, Yusuf S, Devereaux PJ (2008). Cardiovascular sequelae of preeclampsia/eclampsia: a systematic review and meta-analyses. *American Heart Journal*.

[3] Lykke JA, Langhoff-Roos J, Sibai BM, Funai EF, Triche EW, Paidas MJ (2009). Hypertensive pregnancy disorders and subsequent cardiovascular morbidity and type 2 diabetes mellitus in the mother. *Hypertension*.

[4] Mosca L, Benjamin EJ, Berra K (2011). Effectiveness-based guidelines for the prevention of cardiovascular disease in women—2011 update: a Guideline from the American Heart Association. *Circulation*.

[5] Magee LA, von Dadelszen P (2007). Pre-eclampsia and increased cardiovascular risk. *British Medical Journal*.

[6] Irgens HU, Reisæter L, Irgens LM, Lie RT (2001). Long term mortality of mothers and fathers after pre-eclampsia: population based cohort study. *British Medical Journal*.

[7] Lazdam M, De La Horra A, Pitcher A (2010). Elevated blood pressure in offspring born premature to hypertensive pregnancy: is endothelial dysfunction the underlying vascular mechanism?. *Hypertension*.

[8] Higgins M, Keller J, Moore F (1980). Studies of blood pressure in Tecumseh, Michigan. I. Blood pressure in young people and its relationship to personal and familial characteristics and complications of pregnancy in mothers. *The American Journal of Epidemiology*.

[9] Langford HG, Watson RL (1980). Prepregnant blood pressure, hypertension during pregnancy, and later blood pressure of mothers and offspring. *Hypertension*.

[10] Palti H, Rothschild E (1989). Blood pressure and growth at 6 years of age among offsprings of mothers with hypertension of pregnancy. *Early Human Development*.

[11] Seidman DS, Laor A, Gale R, Stevenson DK, Mashiach S, Danon YL (1991). Pre-eclampsia and offspring’s blood pressure, cognitive ability and physical development at 17-years-of-age. *British Journal of Obstetrics & Gynaecology*.

[12] Tenhola S, Rahiala E, Halonen P, Vanninen E, Voutilainen R (2006). Maternal preeclampsia predicts elevated blood pressure in 12-year-old children: evaluation by ambulatory blood pressure monitoring. *Pediatric Research*.

[13] Robb AO, Mills NL, Din JN (2009). Influence of the menstrual cycle, pregnancy, and preeclampsia on arterial stiffness. *Hypertension*.

[14] Yinon Y, Kingdom JCP, Odutayo A (2010). Vascular dysfunction in women with a history of preeclampsia and intrauterine growth restriction: insights into future vascular risk. *Circulation*.

[15] Evans CS, Gooch L, Flotta D (2011). Cardiovascular system during the postpartum state in women with a history of preeclampsia. *Hypertension*.

[16] Páez O, Alfie J, Gorosito M (2009). Parallel decrease in arterial distensibility and in endothelium-dependent dilatation in young women with a history of pre-eclampsia. *Clinical and Experimental Hypertension*.

[17] Elvan-Taşpinar A, Bots ML, Franx A, Bruinse HW, Engelbert RHH (2005). Stiffness of the arterial wall, joints and skin in women with a history of pre-eclampsia. *Journal of Hypertension*.

[18] Lampinen KH, Rönnback M, Kaaja RJ, Groop PH (2006). Impaired vascular dilatation in women with a history of pre-eclampsia. *Journal of Hypertension*.

[19] Blaauw J, van Pampus MG, van Doormaal JJ (2006). Increased intima-media thickness after early-onset preeclampsia. *Obstetrics and Gynecology*.

[20] Kuscu NK, Kurhan Z, Yildirim Y, Tavli T, Koyuncu F (2003). Detection of endothelial dysfunction in preeclamptic patients by using color Doppler sonography. *Archives of Gynecology and Obstetrics*.

[21] Noori M, Donald AE, Angelakopoulou A, Hingorani AD, Williams DJ (2010). Prospective study of placental angiogenic factors and maternal vascular function before and after preeclampsia and gestational hypertension. *Circulation*.

[22] Hamad RR, Eriksson MJ, Silveira A, Hamsten A, Bremme K (2007). Decreased flow-mediated dilation is present 1 year after a pre-eclamptic pregnancy. *Journal of Hypertension*.

[23] Germain AM, Romanik MC, Guerra I (2007). Endothelial dysfunction: a link among preeclampsia, recurrent pregnancy loss, and future cardiovascular events?. *Hypertension*.

[24] Chambers JC, Fusi L, Malik IS, Haskard DO, De Swiet M, Kooner JS (2001). Association of maternal endothelial dysfunction with preeclampsia. *Journal of the American Medical Association*.

[25] Lommerse T, Aardenburg R, Houben A, Peeters LL (2007). Endothelium-dependent vasodilatation in formerly preeclamptic women correlates inversely with body mass index and varies independently of plasma volume. *Reproductive Sciences*.

[26] Agatisa PK, Ness RB, Roberts JM, Costantino JP, Kuller LH, McLaughlin MK (2004). Impairment of endothelial function in women with a history of preeclampsia: an indicator of cardiovascular risk. *American Journal of Physiology—Heart and Circulatory Physiology*.

[27] Kvehaugen AS, Dechend R, Ramstad HB, Troisi R, Fugelseth D, Staff AC (2011). Endothelial function and circulating biomarkers are disturbed in women and children after preeclampsia. *Hypertension*.

[28] Khan F, Belch JJF, MacLeod M, Mires G (2005). Changes in endothelial function precede the clinical disease in women in whom preeclampsia develops. *Hypertension*.

[29] Blaauw J, Graaff R, van Pampus MG (2005). Abnormal endothelium-dependent microvascular reactivity in recently preeclamptic women. *Obstetrics and Gynecology*.

[30] Ramsay JE, Stewart F, Greer IA, Sattar N (2003). Microvascular dysfunction: a link between pre-eclampsia and maternal coronary heart disease. *British Journal of Obstetrics and Gynaecology*.

[31] Steegers EAP, von Dadelszen P, Duvekot JJ, Pijnenborg R (2010). Pre-eclampsia. *The Lancet*.

[32] Redman CWG, Sargent IL (2010). Immunology of pre-eclampsia. *American Journal of Reproductive Immunology*.

[33] Redman CWG, Sargent IL (2009). Placental stress and pre-eclampsia: a revised view. *Placenta*.

[34] Rodie VA, Freeman DJ, Sattar N, Greer IA (2004). Pre-eclampsia and cardiovascular disease: metabolic syndrome of pregnancy?. *Atherosclerosis*.

[35] Poston L (2006). Endothelial dysfunction in pre-eclampsia. *Pharmacological Reports*.

[36] Levine RJ, Lam C, Qian C (2006). Soluble endoglin and other circulating antiangiogenic factors in preeclampsia. *The New England Journal of Medicine*.

[37] Levine RJ, Maynard SE, Qian C (2004). Circulating angiogenic factors and the risk of preeclampsia. *The New England Journal of Medicine*.

[38] Ahmad S, Ahmed A (2004). Elevated placental soluble vascular endothelial growth factor receptor-1 inhibits angiogenesis in preeclampsia. *Circulation Research*.

[39] Wang A, Rana S, Karumanchi SA (2009). Preeclampsia: the role of angiogenic factors in its pathogenesis. *Physiology*.

[40] George EM, Granger JP (2011). Endothelin: key mediator of hypertension in preeclampsia. *American Journal of Hypertension*.

[41] Yoshida A, Nakao S, Kobayashi M, Kobayashi H (2000). Flow-mediated vasodilation and plasma fibronectin levels in preeclampsia. *Hypertension*.

[42] Savvidou MD, Hingorani AD, Tsikas D, Frölich JC, Vallance P, Nicolaides KH (2003). Endothelial dysfunction and raised plasma concentrations of asymmetric dimethylarginine in pregnant women who subsequently develop pre-eclampsia. *The Lancet*.

[43] Nishikawa S, Miyamoto A, Yamamoto H, Ohshika H, Kudo R (2001). Preeclamptic serum enhances endothelin-converting enzyme expression in cultured endothelial cells. *American Journal of Hypertension*.

[44] Knock GA, Poston L (1996). Bradykinin-mediated relaxation of isolated maternal resistance arteries in normal pregnancy and preeclampsia. *American Journal of Obstetrics and Gynecology*.

[45] Cockell AP, Poston L (1997). Flow-mediated vasodilatation is enhanced in normal pregnancy but reduced in preeclampsia. *Hypertension*.

[46] Yamamoto T, Suzuki Y, Kojima K, Suzumori K (2005). Reduced flow-mediated vasodilation is not due to a decrease in production of nitric oxide in preeclampsia. *American Journal of Obstetrics and Gynecology*.

[47] Bowyer L, Brown MA, Jones M (2003). Forearm blood flow in pre-eclampsia. *British Journal of Obstetrics and Gynaecology*.

[48] Anastasakis E, Paraskevas KI, Papantoniou N (2008). Association between abnormal uterine artery Doppler flow velocimetry, risk of preeclampsia, and indices of arterial structure and function: a pilot study. *Angiology*.

[49] Hasan KM, Manyonda IT, Ng FS, Singer DRJ, Antonios TFT (2002). Skin capillary density changes in normal pregnancy and pre-eclampsia. *Journal of Hypertension*.

[50] Sibai B, Dekker G, Kupferminc M (2005). Pre-eclampsia. *The Lancet*.

[51] Poston L, Briley A, Seed P, Kelly F, Shennan A (2006). Vitamin C and vitamin E in pregnant women at risk for pre-eclampsia (VIP trial): randomised placebo-controlled trial. *The Lancet*.

[52] Meher S, Duley L (2007). Nitric oxide for preventing pre-eclampsia and its complications. *Cochrane Database of Systematic Reviews*.

[53] Luscher TF (1994). The endothelium and cardiovascular disease—a complex relation. *The New England Journal of Medicine*.

[54] Halcox JPJ, Schenke WH, Zalos G (2002). Prognostic value of coronary vascular endothelial dysfunction. *Circulation*.

[55] Suwaidi JA, Hamasaki S, Higano ST, Nishimura RA, Holmes DR, Lerman A (2000). Long-term follow-up of patients with mild coronary artery disease and endothelial dysfunction. *Circulation*.

[56] Hadi HA, Carr CS, Al Suwaidi J (2005). Endothelial dysfunction: cardiovascular risk factors, therapy, and outcome. *Vascular Health and Risk Management*.

[57] Leeson CPM, Kattenhorn M, Morley R, Lucas A, Deanfield JE (2001). Impact of low birth weight and cardiovascular risk factors on endothelial function in early adult life. *Circulation*.

[58] McEniery CM, Qasem A, Schmitt M, Avolio AP, Cockcroft JR, Wilkinson IB (2003). Endothelin-1 regulates arterial pulse wave velocity in vivo. *Journal of the American College of Cardiology*.

[59] Wilkinson IB, Qasem A, McEniery CM, Webb DJ, Avolio AP, Cockcroft JR (2002). Nitric oxide regulates local arterial distensibility in vivo. *Circulation*.

[60] McEniery CM, Wallace S, MacKenzie IS (2006). Endothelial function is associated with pulse pressure, pulse wave velocity, and augmentation index in healthy humans. *Hypertension*.

[61] Halcox JPJ, Donald AE, Ellins E (2009). Endothelial function predicts progression of carotid intima-media thickness. *Circulation*.

[62] Juonala M, Viikari JSA, Laitinen T (2004). Interrelations between brachial endothelial function and carotid intima-media thickness in young adults: the Cardiovascular Risk in Young Finns Study. *Circulation*.

[63] Touboul PJ, Hennerici MG, Meairs S (2004). Mannheim intima-media thickness consensus. *Cerebrovascular Diseases*.

[64] Sanada S, Node K, Minamino T (2003). Long-acting Ca^2+^ blockers prevent myocardial remodeling induced by chronic NO inhibition in rats. *Hypertension*.

[65] Barouch LA, Harrison RW, Skaf MW (2002). Nitric oxide regulates the heart by spatial confinement of nitric oxide synthase isoforms. *Nature*.

[66] Levy D, Garrison RJ, Savage DD, Kannel WB, Castelli WP (1990). Prognostic implications of echocardiographically determined left ventricular mass in the Framingham Heart Study. *The New England Journal of Medicine*.

[67] Perticone F, Maio R, Ceravolo R, Cosco C, Cloro C, Mattioli PL (1999). Relationship between left ventricular mass and endothelium-dependent vasodilation in never-treated hypertensive patients. *Circulation*.

[68] Sung J, Ouyang P, Bacher AC (2002). Peripheral endothelium-dependent flow-mediated vasodilatation is associated with left ventricular mass in older persons with hypertension. *American Heart Journal*.

[69] Palmieri V, Storto G, Arezzi E (2005). Relations of left ventricular mass and systolic function to endothelial function and coronary flow reserve in healthy, new discovered hypertensive subjects. *Journal of Human Hypertension*.

[70] Lind L (2008). Left ventricular mass is related to endothelium-dependent vasodilation in the forearm, but not in the brachial artery, in elderly subjects: the Prospective Investigation of the Vasculature in Uppsala Seniors study. *Journal of Human Hypertension*.

[71] Hasegawa T, Boden-Albala B, Eguchi K (2010). Impaired flow-mediated vasodilatation is associated with increased left ventricular mass in a multiethnic population. the northern manhattan study. *American Journal of Hypertension*.

[72] Yeboah J, Crouse JR, Bluemke DA (2011). Endothelial dysfunction is associated with left ventricular mass (assessed using MRI) in an adult population (MESA). *Journal of Human Hypertension*.

[73] Antoniades C, Mussa S, Shirodaria C (2009). Relation of preoperative radial artery flow-mediated dilatation to nitric oxide bioavailability in radial artery grafts used in off-pump coronary artery bypass grafting. *The American Journal of Cardiology*.

[74] Leeson C, Robinson M, Francis J (2006). Cardiovascular magnetic resonance imaging for non-invasive assessment of vascular function: validation against ultrasound. *Journal of Cardiovascular Magnetic Resonance*.

[75] Urbina EM, Williams RV, Alpert BS (2009). Noninvasive assessment of subclinical atherosclerosis in children and adolescents: recommendations for standard assessment for clinical research: a scientific statement from the american heart association. *Hypertension*.

[76] Lorenz MW, Markus HS, Bots ML, Rosvall M, Sitzer M (2007). Prediction of clinical cardiovascular events with carotid intima-media thickness: a systematic review and meta-analysis. *Circulation*.

[77] Ajne G, Wolff K, Fyhrquist F, Carlström K, Hemsén-Mörtberg A, Nisell H (2003). Endothelin converting enzyme (ECE) activity in normal pregnancy and preeclampsia. *Hypertension in Pregnancy*.

[78] Stewart FM, Freeman DJ, Ramsay JE, Greer IA, Caslake M, Ferrell WR (2007). Longitudinal assessment of maternal endothelial function and markers of inflammation and placental function throughout pregnancy in lean and obese mothers. *Journal of Clinical Endocrinology and Metabolism*.

[79] Freeman DJ, McManus F, Brown EA (2004). Short- and long-term changes in plasma inflammatory markers associated with preeclampsia. *Hypertension*.

[80] Sattar N, Ramsay J, Crawford L, Cheyne H, Greer IA (2003). Classic and novel risk factor parameters in women with a history of preeclampsia. *Hypertension*.

[81] Houben AJHM, De Leeuw PW, Peeters LLH (2007). Configuration of the microcirculation in pre-eclampsia: possible role of the venular system. *Journal of Hypertension*.

[82] Kajantie E, Eriksson JG, Osmond C, Thornburg K, Barker DJP (2009). Pre-eclampsia is associated with increased risk of stroke in the adult offspring the helsinki birth cohort study. *Stroke*.

[83] Palmsten K, Buka SL, Michels KB (2010). Maternal pregnancy-related hypertension and risk for hypertension in offspring later in life. *Obstetrics and Gynecology*.

[84] Ferreira I, Peeters LL, Stehouwer CDA (2009). Preeclampsia and increased blood pressure in the offspring: meta-analysis and critical review of the evidence. *Journal of Hypertension*.

[85] NICE (2011). Hypertension (Update). http://www.nice.org.uk/newsroom/pressreleases/NewGuidelineForDiagnosingAndTreatingHighBloodPressure.jsp.

[86] Akcakus M, Altunay L, Yikilmaz A, Yazici C, Koklu E (2010). The relationship between abdominal aortic intima-media thickness and lipid profile in neonates born to mothers with preeclampsia. *Journal of Pediatric Endocrinology and Metabolism*.

[87] Jayet PY, Rimoldi SF, Stuber T (2010). Pulmonary and systemic vascular dysfunction in young offspring of mothers with preeclampsia. *Circulation*.

[88] Leeson CPM, Whincup PH, Cook DG (1997). Flow-mediated dilation in 9- to 11-year-old children: the influence of intrauterine and childhood factors. *Circulation*.

[89] Fowden AL, Giussani DA, Forhead AJ (2006). Intrauterine programming of physiological systems: causes and consequences. *Physiology*.

[90] Catarino C, Rebelo I, Belo L (2009). Fetal and maternal angiogenic/anti-angiogenic factors in normal and preeclamptic pregnancy. *Growth Factors*.

[91] Braekke K, Ueland PM, Harsem NK, Staff AC (2009). Asymmetric dimethylarginine in the maternal and fetal circulation in preeclampsia Fetal and maternal angiogenic/anti-angiogenic factors in normal and preeclamptic pregnancy. *Pediatric Research*.

[92] Leeson P (2007). Pediatric prevention of atherosclerosis: targeting early variation in vascular biology. *Pediatrics*.

